# Does attention matter more than exposure? A pilot RCT of nature-based mindfulness for mental well-being and stress

**DOI:** 10.3389/fpsyg.2026.1905841

**Published:** 2026-09-24

**Authors:** Sabine V. Huemer, Zhirui Hu, Makena Aiona-Agra

**Affiliations:** 1 NEST Lab, School of Psychological Science, Oregon State University, Corvallis, OR, United States; 2 Zhirui’s Lab, Department of Statistics, Oregon State University, Corvallis, OR, United States

**Keywords:** mental well-being, mindfulness, nature connectedness, nature exposure, pilot study, randomized controlled trial, stress reduction

## Abstract

Contact with nature supports psychological health, yet whether the quality of mindful engagement matters more than the quantity of exposure remains unclear. This pilot randomized controlled trial examined a brief, structured nature-based mindfulness program’s effects on mental well-being, perceived stress, and nature connectedness relative to an unstructured outdoor walking control. Participants (*N* = 35) were randomly assigned to daily 15-min guided nature-based mindfulness sessions or daily unstructured outdoor walks over 2 weeks. Point estimates favored the intervention group for mental well-being and perceived stress, but neither group difference reached statistical significance, and the sample was well below what would be needed to detect effects of this magnitude with adequate power. Participants with lower baseline mental well-being achieved greater gains in the intervention group than participants with higher baseline well-being, an advantage not observed in the control group. Both groups increased in nature connectedness with no significant between-group difference; mediation analyses found no evidence that connectedness mediated intervention effects. The intervention group achieved these outcomes despite significantly less total nature exposure than controls, though duration was not independently manipulated. These preliminary findings may serve as hypothesis-generating for future, adequately powered trials.

## 1 Introduction

Psychological and public health research has established that contact with nature supports mental well-being, reduces stress, and promotes psychological flourishing (Martin et al., 2020; Ma et al., 2024; Ulrich, 1983). Two complementary theoretical frameworks guide this work: Ulrich (1983) Stress Reduction Theory (SRT), which emphasizes affective and physiological recovery through nature exposure, and Kaplan and Kaplan (1989) Attention Restoration Theory (ART), which focuses on cognitive restoration through attentional engagement with natural settings. Recent research confirms these restorative effects across stress reduction, emotion regulation, and attentional recovery (Baceviciene and Jankauskiene, 2022; Bakir-Demir et al., 2021; Olszewska-Guizzo et al., 2022), with benefits closely linked to nature connectedness, an emotional relationship with the natural world associated with psychological well-being outcomes (Barragan-Jason et al., 2022; Martin et al., 2020).

However, much of this literature treats nature exposure as a largely unidimensional construct indexed by duration or frequency, with comparatively less attention to the quality of engagement during that exposure. A growing body of work suggests that how people attend to nature may matter more than how long they are in it. Richardson et al. (2021) found that brief, active noticing of nature predicted well-being more strongly than simple time spent outdoors, and earlier experimental work supports this pattern: Passmore and Holder (2017) showed that participants instructed to notice how everyday nature made them feel reported significantly higher well-being than a business-as-usual control group despite no increase in time spent outdoors. McEwan et al. (2019) similarly found that a brief smartphone-delivered intervention prompting users to notice good things in urban nature produced significant gains in well-being and nature connectedness over 1 week. Most directly relevant to the present design, Nisbet et al. (2019) randomly assigned participants to a 20-min outdoor walk preceded by either a brief mindfulness instruction or no instruction; despite identical exposure duration, the mindfulness-primed group reported significantly less negative affect and greater nature connectedness, offering direct evidence that instructional framing, not exposure itself, shaped affective outcomes. More recently, Giusti et al. (2025) found that guided forest-bathing sessions helped participants form new, more relational ways of engaging with nature that unguided time outdoors did not foster. Taken together, this literature suggests that quality of engagement, not quantity of exposure, may be the more proximal determinant of nature’s psychological benefits. However, few studies have paired a quality manipulation (i.e., structured instruction) with an active, duration-matched nature control condition across a multi-day intervention, leaving open how a sustained, real-world instructional program compares to unstructured nature engagement over time.

Emerging work demonstrates that mindfulness practices in natural settings can amplify these restorative processes (Choe et al., 2020; Djernis et al., 2019; Nisbet et al., 2019). Mindfulness, defined as the intentional, nonjudgmental awareness of the present moment, shares conceptual overlap with both SRT and ART: it reduces physiological stress reactivity and enhances attentional regulation (Creswell, 2017; Tang et al., 2015). When practiced outdoors, mindfulness may therefore compound the restorative effects of both theories by simultaneously targeting affective and cognitive recovery systems. Meta-analytic evidence shows that outdoor mindfulness enhances mood and deepens nature connectedness relative to indoor practice, with even brief interventions producing meaningful improvements (Choe et al., 2020; Djernis et al., 2019; Burdick and Camhi, 2024; Ibes and Forestell, 2022). A recent narrative review of nature-based mindfulness interventions found consistent reduction in stress, anxiety, and depressive symptoms alongside improvements in vitality and self-regulation across heterogeneous study designs (Vecchio et al., 2026). Unstructured outdoor walking is itself associated with stress reduction and mood improvement independent of mindfulness (Ma et al., 2024), making it a theoretically active and more conservative comparison condition than a sedentary or indoor control.

Nature connectedness, defined as an individual’s felt sense of emotional and cognitive affiliation with the natural world (Mayer and Frantz, 2004), is a multidimensional construct, encompassing material, experiential, cognitive, emotional, and philosophical forms of connection to nature (Ives et al., 2017). Clarifying which of these dimensions a given nature-based intervention engages, rather than assuming exposure alone builds connectedness, is central to understanding when and why such interventions support well-being.

The present study reports a randomized controlled pilot trial of a structured, nature-based mindfulness intervention that treats nature as an active partner rather than a backdrop. The program was designed to be practical and accessible, offering brief guided activities requiring no equipment and completable in any nearby green space with trees. This study examines the program’s effectiveness in enhancing mental well-being, strengthening nature connectedness, and reducing stress relative to walking alone, contributing to the expanding evidence base for nature-based mindfulness interventions (Burdick and Camhi, 2024; Joschko et al., 2023; Racz et al., 2024; Vecchio et al., 2026).

The present intervention was informed by both SRT and ART, though it was not designed to directly test either theory’s proposed mechanisms. Well-being improvement and momentary stress reduction were selected as primary outcomes consistent with SRT’s affective recovery model. Although ART’s proposed cognitive mechanisms are theoretically relevant, the present study did not include attentional measures and therefore cannot speak to them directly.

*Primary hypotheses*: Participants who engaged in daily 15-min guided nature-based mindfulness practice over 2 weeks were expected to exhibit significantly higher levels of mental well-being (H1) and significantly lower levels of perceived stress (H2) compared to participants who completed daily unstructured outdoor walks.

*Secondary hypotheses*: Nature connectedness was expected to increase significantly in the intervention group, with greater gains observed than in the control group (H3a). Furthermore, post-intervention nature connectedness was expected to predict post-intervention mental well-being and perceived stress across both groups after controlling for baseline levels (H3b), with higher connectedness associated with higher well-being and lower stress.

## 2 Methods and materials

### 2.1 Participants

A total of 179 individuals completed prescreening; 141 consented to participate in this remotely delivered two-week study. Of these, 103 completed the intake questionnaire and were randomized to condition. Of these, 35 (16 intervention, 19 control) completed the full trial and exit questionnaire, comprising the final analytic sample (see Figure 1). Daily practice log data used in the exploratory analyses were available for 32 of these 35 participants.

**Figure 1:**
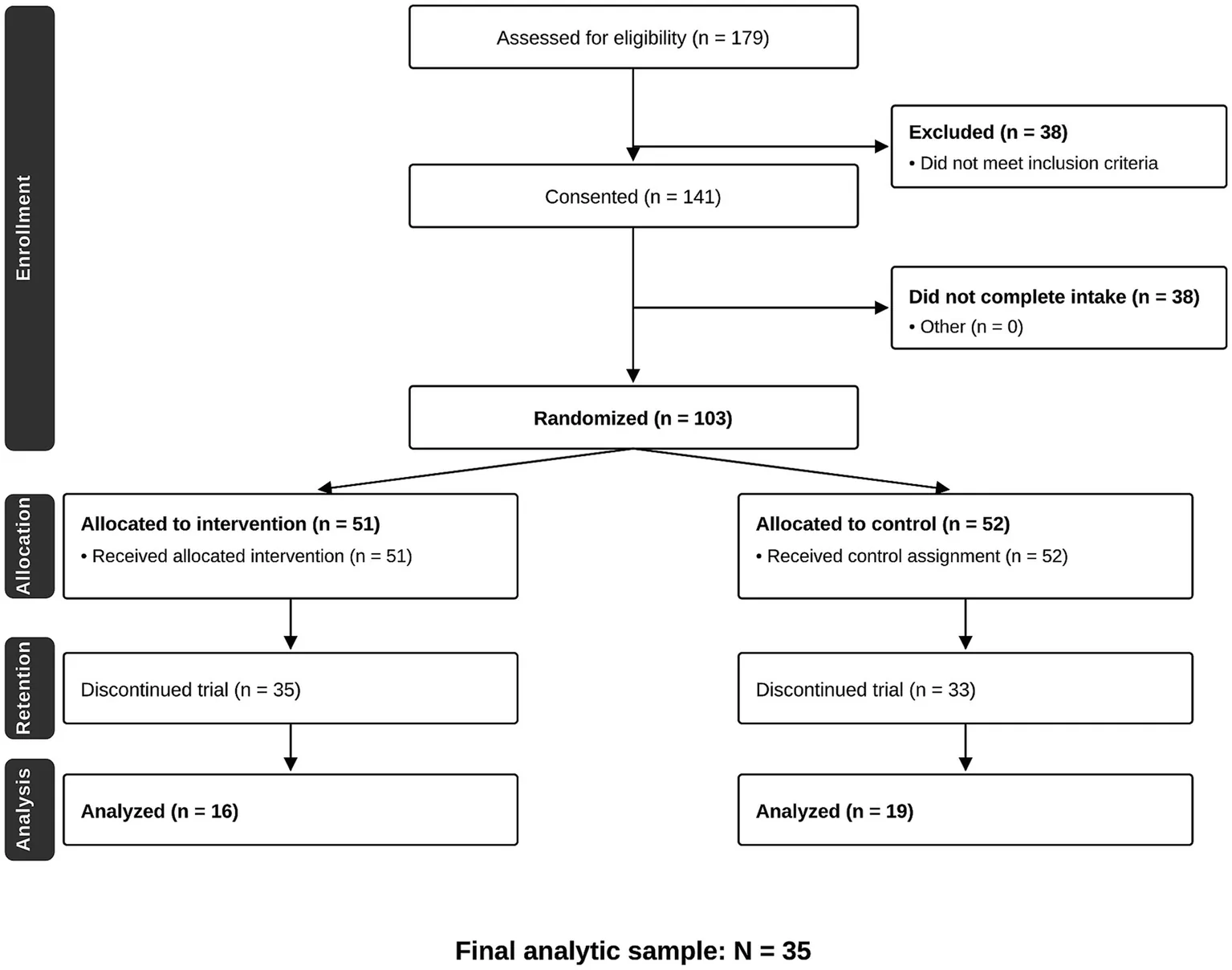
Flow of participants through the trial. CONSORT flow diagram illustrating participant screening, randomization, retention, and final analytic sample.

Participants ranged in age from 18 to 72 years (*M* = 46.32, *SD* = 17.30). Ethnic identity was reported as: 80.0% Caucasian, 8.6% Asian, 2.9% Hispanic, and 8.6% not reported. Gender identity was 74.3% female, 14.3% male, 5.7% transgender, and 5.7% non-binary (see Appendix Table A1).

Attrition in the present study is consistent with patterns observed in remote, self-guided digital mindfulness interventions, which typically show higher dropout than guided or compensated designs (Linardon, 2023). The primary purpose of pilot randomized controlled trials is to establish feasibility and generate preliminary effect estimates to inform a future, adequately powered trial, rather than to test hypotheses at conventional power thresholds (Lakens, 2022). We justify our sample size using established pilot-study conventions rather than an after-the-fact power estimates. Our final sample size (*N* = 35; *n* = 16 and *n* = 19 per group) exceeds the commonly cited minimum of 12 participants per group for early-phase pilot trials (Julious, 2005), though it falls below the more conservative benchmark of approximately 35 per group recommended for precise estimation of outcome variability (Teare et al., 2014; Whitehead et al., 2016), a limitation we acknowledge. Completers (*n* = 35) did not differ significantly from non-completers (*n* = 68) on age, gender, ethnicity, perceived stress, trait mindfulness, or nature connectedness (all *p*s > 0.15, Appendix Table A2).

Participants were recruited via campus postcards, Oregon State University Extension mailing lists, and social media. This reflects a combination of convenience and snowball sampling, as some participants also learned of the study through personal contacts who had already enrolled. The majority practiced in Oregon (*n* = 22, 62.9%); additional participants were located in the southeastern United States (*n* = 6), other U.S. states (*n* = 5), and internationally (*n* = 2). Data were collected between May and August 2025.

#### 2.1.1 Ethics statement

This study was approved by the Oregon State University Institutional Review Board (IRB # HE-2025-1492). Written informed consent was obtained from all individual participants included in the study; all participants were over 18 years of age. Participants provided written informed consent for their anonymized data to be published.

### 2.2 Materials and measures

#### 2.2.1 Training materials

All participants viewed a brief (5-min) training video prior to beginning the study, which explained the logistics of this fully remote, self-paced protocol: participants were free to choose when and where to practice each day (for the intervention group, a nearby green space, ideally including a tree, was suggested), how to complete the daily practice log using either the provided printable form or an equivalent personal record with the same fields, and the process for submitting logs and completing the end-of-study exit questionnaire, which mirrored the intake measures already completed at baseline.

The intervention group additionally received two distinct 15-min guided practice sequences, each available in both audio and video format (see Appendix B for script excerpts). Both sequences followed the same overall progression, from bodily slowing and grounding to multisensory immersion paired with breathing, to a closing segment inviting participants to view their natural surroundings with a sense of awe and playful appreciation. Individual segments lasted 1 to 4 min each. The two sequences were designed to produce a comparable overall experience while varying in specific content and imagery, to reduce repetitiveness across the 14-day protocol; participants could select either sequence on any given day. Participants were encouraged to use the video version for their first practice and were free to use the audio-only version thereafter; guidance throughout was intentionally brief and low-key, intended to support participants’ attention toward the natural environment rather than the recording itself. Headphone, earbud or speaker use was left to the participants’ discretion.

#### 2.2.2 Warwick–Edinburgh mental well-being scale (WEMWBS)

Mental well-being was assessed at the beginning and end of the intervention using the 14-item WEMWBS (Tennant et al., 2007), rated 1 (*none of the time*) to 5 (*all of the time*). Internal consistency was excellent at both pre-test (*α* = 0.90) and post-test (*α* = 0.89).

#### 2.2.3 Perceived stress scale (PSS-10)

Perceived stress was measured at the beginning and end of the study using the 10-item PSS-10 (Cohen and Williamson, 1988), administered using a 1 (*never*) to 5 (*very often*) response format, rather than the standard 0 (*never*) to 4 (*very often*) format, so that response options were presented consistently across all study measures (WEMWBS, FFMQ-SF, and CNS each used a 1-to-5 format). We converted the values to standard 0–40 in the analysis. Internal consistency was good at pre-test (*α* = 0.86) and post-test (*α* = 0.85).

#### 2.2.4 Five facet mindfulness questionnaire—short form (FFMQ-SF)

Trait mindfulness was assessed using the 24-item FFMQ-SF (Bohlmeijer et al., 2011), a short-form adaptation of Baer et al.'s (2006) Five Facet Mindfulness Questionnaire, which conceptualizes mindfulness as comprising five facets: observing, describing, acting with awareness, nonjudging of inner experience, and nonreactivity to inner experience. Items were rated 1 (*never or very rarely true*) to 5 (*very often or always true*). Internal consistency was good at pre-test (*α* = 0.75) and post-test (*α* = 0.88).

#### 2.2.5 Connectedness to nature scale (CNS)

Nature connectedness was assessed using items from the CNS (Mayer and Frantz, 2004), scored 1 (*strongly disagree*) to 5 (*strongly agree*). Internal consistency was good at pre-test (*α* = 0.71) and post-test (*α* = 0.76).

#### 2.2.6 Daily practice log

Participants recorded pre and post-practice stress ratings (1–10 scale), total minutes practiced, practice location, and contextual notes each day. The intervention group additionally reported which guided practice sequence was used.

### 2.3 Procedure

After completing eligibility screening, consent, and intake measures, participants were randomly assigned using an online true random number generator (Random.org; Haahr, 1998). Intervention participants were guided by a fixed 15-min audio or video sequence, while control participants were instructed to walk outdoors for a target duration of approximately 15 min. As this was a fully remote, self-paced protocol, neither condition’s duration was monitored in real time: control participants’ walks were not bounded by a structured end point or guided pacing and may in practice have extended beyond the target duration, and intervention participants were required to travel to a practice location (e.g., a nearby green space) before beginning their guided session, with travel time not systematically distinguished from practice time in participants’ self-reports. Daily practice duration for both groups was self-reported via the daily practice log, with participants recording what they personally considered their practice time. At the end of the study, participants completed an exit questionnaire mirroring the intake measures and provided qualitative feedback. Control participants were given access to the guided mindfulness materials following study completion. Participants who completed the study were entered into a raffle for six $25 gift cards.

### 2.4 Data analysis

All analyses were conducted in R (version 4.2.1; R Core Team, 2022). Linear mixed-effects models were fitted using the lme4 package (Bates et al., 2015) and mediation analyses using the mediation package (Tingley et al., 2014). Baseline equivalence was verified using independent-samples t-tests and chi-square tests. The alpha level was set at 0.05 for all analyses. The effect estimates with 95% confidence intervals are reported.

To test Hypotheses 1, 2, and 3a, separate linear mixed-effects models were fitted for each outcome (mental well-being, perceived stress, and nature connectedness), regressing each outcome on time (pre-vs. post-intervention), experimental group, and their Time × Group interaction, with a random intercept for each participant. The significance of each Time × Group interaction was evaluated using a likelihood ratio test (LRT) comparing the full model against a reduced model without the interaction term. A reduced model without interaction term testing the main effect of time was also fitted. Effect sizes for pre-to-post changes were estimated using Hedges’ g, which corrects for positive bias in small samples (Hedges, 1981). Confidence intervals for the mixed-model fixed effects were obtained by parametric bootstrap; intervals are percentile intervals based on 5,000 replicates. Confidence intervals for Hedges’ g are noncentral t intervals.

Because this was a pilot trial, no *a priori* power analysis was conducted and the sample size was determined by recruitment feasibility. To inform the design of a future definitive trial, we therefore conducted a prospective, simulation-based sample size projection for the Time × Group interaction using the simr package (Green and MacLeod, 2016). Power was simulated from the fitted linear mixed-effects models, so that the projection incorporated the estimated between-participant and residual variances as well as the fixed effects; the interaction was tested by likelihood ratio test at *α* = 0.05, with 1,000 simulations at each candidate sample size. Sensitivity analyses for primary outcomes included baseline covariate adjustment (age, nature exposure, pre-intervention scores on relevant outcomes, trait mindfulness, and CNS), added in the model simultaneously (Supplementary Table S1).

For Hypothesis 3b, mediation models were fitted with post-intervention PSS and MWB as outcomes, post-intervention CNS as the mediator, and experimental group as the treatment variable, including baseline covariates in both the mediator and outcome models. Confidence intervals were estimated using nonparametric bootstrapping using percentile intervals (5,000 resamples). Sensitivity analyses examined robustness to unmeasured mediator–outcome confounding by varying the assumed residual correlation between models.

Intent-to-treat analyses. The intent-to-treat population comprised all randomized participants for whom baseline data were available. Each outcome was analyzed with a linear mixed-effects model regressing the outcome on time, allocation, and their interaction, with a random intercept for participant, estimated by maximum likelihood. The same model was fitted three ways: observed data only; all randomized participants by full-information maximum likelihood, which incorporates the observed baselines of non-completers without imputing outcomes; and under multiple imputation, in which 500 completed datasets were generated under a missing-at-random assumption, the identical mixed model was fitted to each, and estimates were pooled by Rubin’s rules. Mice package is used for imputation.

Moderation by baseline well-being. Moderation was assessed by adding the Baseline × Time × Group interaction to the same mixed model, with baseline mean-centered, and testing it by likelihood-ratio test against the otherwise identical model omitting the three-way interaction term. We additionally conducted a simulation-based sensitivity analysis in which post-intervention scores were generated from the fitted model across a range of hypothetical moderation slopes, holding the observed baselines, allocation ratio and residual variance fixed, and each replicate was analyzed by the same likelihood-ratio test. This identifies the smallest slope the design could detect with 80% power.

## 3 Results

### 3.1 Hypothesis 1: mental well-being

A linear mixed-effects model with a Time × Group interaction estimated group difference in pre-to-post change was 2.63 points, [95% CI (−0.94, 6.10), *p* = 0.126], in favor of the intervention group. The point estimate numerically favored the intervention group, but the interaction did not reach statistical significance, as the available sample falls short of what would be needed to detect an effect of this magnitude reliably (see Figure 2A). The variance components of the fitted model were *τ*^2^ = 37.65 for between-subject variance and *σ*^2^ = 13.14 for residual variance. The model yielded an adjusted ICC of 0.741 and an unadjusted (conditional) ICC of 0.679, An intercept-only model with the same random-effects structure yielded an unconditional ICC of 0.676. The substantial within-subject correlation of the repeated observations supports the inclusion of a participant-level random intercept. Detecting an interaction of the magnitude observed here with 80% power would require approximately 126 participants.

**Figure 2:**
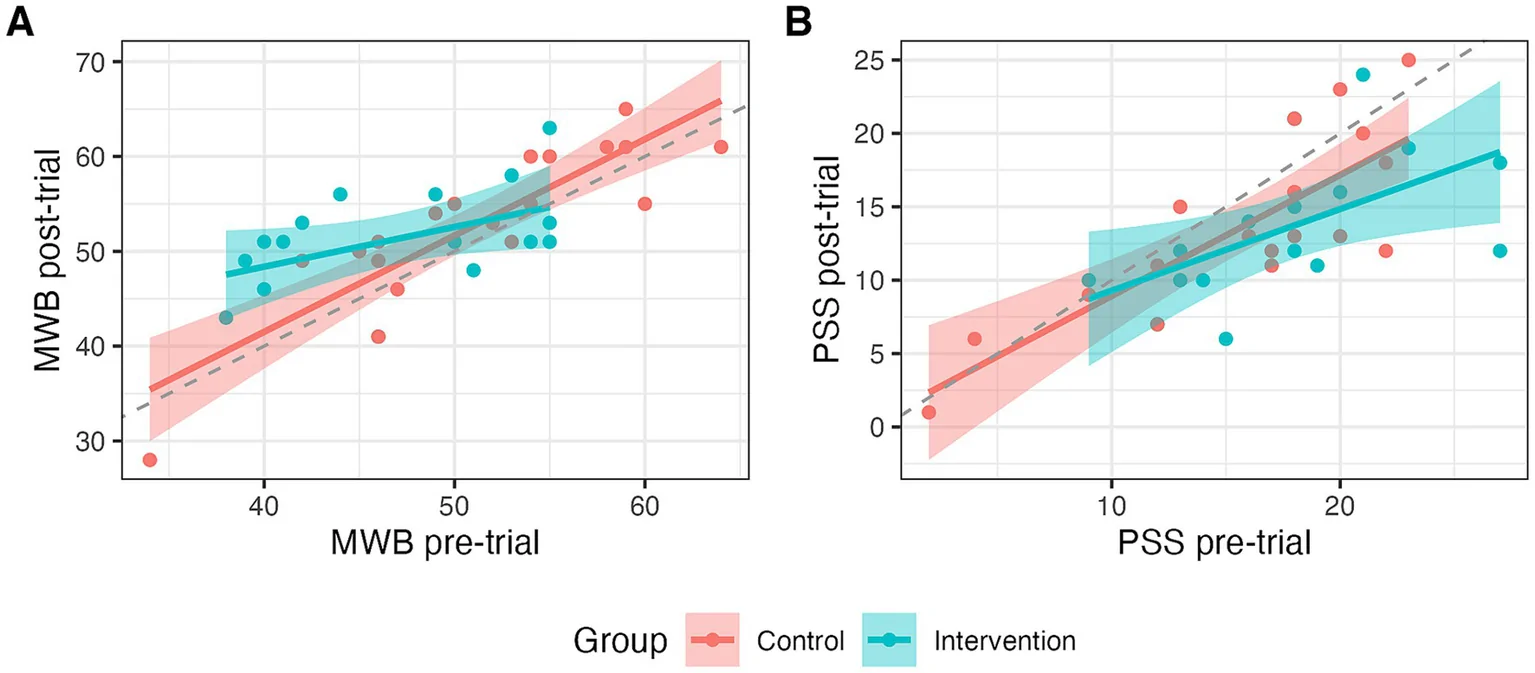
Mental well-being score and perceived stress pre and post-trial. Scatterplot depicting individual participant scores at pre-test and post-test for **(A)** mental well-being (WEMWBS) and **(B)** perceived stress (PSS). The dashed line represents no change (identical pre and post-test scores); points above the line indicate improvement in well-being and below the line indicate reduction in perceived stress. The shaded area represents the 95% confidence interval around the model-predicted regression line. Both groups showed improvement in mental well-being and reduction in perceived stress for most participants. The intervention group’s average change was numerically larger for both outcomes, though neither between-group difference reached statistical significance.

Both the intervention and the control group’s mean MWB showed increases (see Tables A3, A4). Post-intervention MWB was higher in the intervention group across the range of baseline scores, with the advantage most pronounced among participants with lower initial well-being (Figure 2). To test whether the intervention effect on mental well-being depended on initial well-being, the Baseline × Time × Group interaction was added to the primary mixed-effects model. The interaction was significant, *χ*^2^(1) = 6.80, *p* = 0.009, with an estimated slope of −0.60. The intervention effect was therefore larger among participants who began the study with lower well-being. A simulation-based sensitivity analysis indicated that the design had approximately 74% power to detect a slope of this magnitude, with an empirical Type I error rate of 0.045 at a true slope of zero. Because this analysis was not preregistered and rests on 35 participants, we interpret it as exploratory and as a hypothesis for confirmatory testing in an adequately powered trial.

The sensitivity analysis including baseline covariates yielded a consistent magnitude and direction, with the Time × Group interaction remaining positive but not significant, supporting that the primary estimate is not an artefact of baseline imbalance (Supplementary Table S1). Refitting the Time × Group model to all 103 randomized participants with baseline data attenuated primary estimates. For mental well-being, the estimated group difference in pre-to-post change falls to 1.96 points, 95% CI [−1.24, 5.16], under full-information maximum likelihood and 1.50 points, 95% CI [−2.16, 5.15], under multiple imputation (Appendix Table A5).

### 3.2 Hypothesis 2: perceived stress

A linear mixed-effects model with Time × Group interaction for perceived stress estimated group difference in pre-to-post change was −2.02 PSS points [95% CI (−4.84, 0.88), *p* = 0.147], with the point estimate numerically favoring greater stress reduction in the intervention group, though this between-group difference did not reach statistical significance (see Figure 2B). The variance components of the fitted model were τ^2^ = 22.28 for between-subject variance and σ^2^ = 8.66 for residual variance. The model yielded an adjusted ICC of 0.720 and an unadjusted (conditional) ICC of 0.662; an intercept-only model with the same random-effects structure yielded an unconditional ICC of 0.594. As with mental well-being, this level of within-person correlation justifies the participant-level random intercept. Detecting an interaction of the magnitude observed here with 80% power would require approximately 139 participants for perceived stress. Mean perceived stress decreased in both the control group and intervention group, but to a larger extent in the intervention group (see Appendix Tables A3, A4). The sensitivity analysis including baseline covariates yielded consistent estimates in both magnitude and significance, supporting the robustness of the primary finding (Supplementary Table S1). When the Time × Group model was refit to all 103 randomized participants with baseline data, primary estimates were attenuated. For perceived stress, the estimated group difference in pre-to-post change fell to −1.63, 95% CI [−4.21, 0.95], under full-information maximum likelihood and −1.35, 95% CI [−4.22, 1.52], under multiple imputation (Appendix Table A5).

### 3.3 Hypothesis 3a: nature connectedness as outcome

The Time × Group interaction for nature connectedness was not significant, *b* = −1.07, 95% CI [−4.38, 2.33], *p* = 0.508, indicating that the two groups did not differ in magnitude of CNS change. The variance components of the fitted model were τ^2^ = 23.52 for between-subject variance and σ^2^ = 11.94 for residual variance. The model yielded an adjusted ICC of 0.663 and an unadjusted (conditional) ICC of 0.587; an intercept-only model with the same random-effects structure yielded an unconditional ICC of 0.485. This pattern again reflects strong within-person correlation, consistent with the random-intercept specification used throughout. Both groups increased in nature connectedness; combined pre-to-post change was 4.14 CNS points, 95% bootstrap CI [2.53, 5.79]. The main effect of time was significant in the reduced model without the interaction term (*p* < 0.001). The sensitivity analysis including baseline covariates yielded consistent estimates (Supplementary Table S1). When the Time × Group model was refit to all 103 randomized participants with baseline data, the estimated group difference in pre-to-post change was −1.21, 95% CI [−4.33, 1.92], under full-information maximum likelihood and −1.75, 95% CI [−5.31, 1.80], under multiple imputation (see Appendix Table A5).

### 3.4 Hypothesis 3b: nature connectedness as mediator

Although nature connectedness as a mediator was specified as *a priori* as Hypothesis 3b, these analyses are best interpreted as exploratory rather than as a confirmatory test. Internal consistency of the CNS was acceptable in the present sample (*α* = 0.71 at pre-test, 0.76 at post-test), but the small sample and the absence of a group difference in nature connectedness limit what the mediation models can establish.

Mediation analysis examined whether post-intervention CNS mediated the effect of group assignment on post-intervention PSS. Neither the total effect of the intervention on PSS, c = −2.11, 95% CI [−4.33, 0.65], *p* = 0.136, nor the direct effect controlling for CNS, c′ = −2.20, 95% CI [−4.77, 0.61], *p* = 0.124, was statistically significant. The indirect effect via CNS was near zero and non-significant, ACME = 0.10, 95% CI [−0.97, 1.81], *p* = 0.779, as was the proportion mediated, −0.05, 95% CI [−2.15, 1.41], *p* = 0.834, providing no evidence for mediation. In the parallel model with MWB as the outcome, none of the total, 2.46, 95% CI [−1.65, 6.55], *p* = 0.282, direct, 2.66, 95% CI [−1.03, 6.83], *p* = 0.188, or indirect effects, ACME = −0.20, 95% CI [−2.41, 1.41], *p* = 0.724, reached significance. Because the estimated indirect effects were already indistinguishable from zero, sensitivity analysis found them highly fragile to unmeasured confounding; the direct effects, by contrast, remained robust across the full plausible range of confounding examined (Supplementary Table S2). Given the small sample, these null mediation findings are best treated as preliminary, and as providing no positive evidence either for or against a connectedness pathway. Contrary to the anticipated pattern, the two groups did not diverge on either measure. Both increased in nature connectedness (Hedges’ g = 0.58 intervention, 1.11 control) and both decreased in perceived stress (*g* = −0.83 and −0.55, respectively), with no significant Time × Group interaction for either outcome. Both forms of time outdoors were associated with similar improvement in nature connectedness and stress, suggesting the two outcomes rise and fall together.

### 3.5 Exploratory analyses

#### 3.5.1 Daily practice log analyses

As an exploratory, non-preregistered analysis, we examined the association between PSS-10 scores and daily log stress ratings (*n* = 32 with complete data on both). The two measures were substantially associated in levels: PSS-10 scores predicted average daily pre-practice ratings at baseline, *b* = 0.19, *t*(30) = 4.29, *p* < 0.001, *R*^2^ = 0.38; at post-test, *b* = 0.11, *t*(30) = 3.02, *p* = 0.005, *R*^2^ = 0.23; and when both were averaged across the trial, *b* = 0.17, *t*(30) = 4.22, *p* < 0.001, *R*^2^ = 0.37. Change scores, however, were unrelated: change in PSS-10 did not predict change in average daily stress rating, *b* = −0.03, *t*(30) = −0.91, *p* = 0.369, *R*^2^ = 0.03. The two instruments therefore appear to index a common underlying stress level while being sensitive to different sources of variation over time, consistent with the PSS-10 assessing cumulative perceived stress over the past month and the daily log capturing momentary, session-level stress. The daily rating was not independently validated, so a genuine divergence between the two measures cannot be distinguished from attenuation due to its unreliability.

Daily within-session stress reduction (post-practice minus pre-practice rating) was compared between groups in a further exploratory analysis. In a linear mixed-effects model of post-practice stress with pre-practice stress as a covariate and a random intercept for participant (406 observations from 32 participants), the group effect was small and non-significant, *b* = −0.10, SE = 0.26, *t*(30.02) = −0.40, *p* = 0.693, 95% bootstrap CI [−0.59, 0.39]. However, the duration of the practice session predicts within-session stress reduction: each additional minute was associated with a further reduction in post-practice stress, b = −0.006, SE = 0.002, *p* = 0.002 (likelihood ratio test, *χ*^2^(1) = 9.73, *p* = 0.002), and this association did not differ between groups (group × minutes, *b* = −0.009, *p* = 0.137). We therefore do not interpret the session-level difference as evidence of a group advantage.

Averaged across both groups, within-session stress reduction was 1.56 points in the early phase (Days 1–4), 1.56 points in the middle phase (Days 5–9), and 1.39 points in the late phase (Days 10–14; see Figure 3). This three-phase division was a post-hoc, approximately equally spaced descriptive grouping of the 14 days rather than one grounded in a specific hypothesis about habituation or practice effects; no formal statistical comparisons were conducted across phases, and the phase means are not stratified by group. Figure 3 and the phase means should therefore be read as descriptive only.

**Figure 3:**
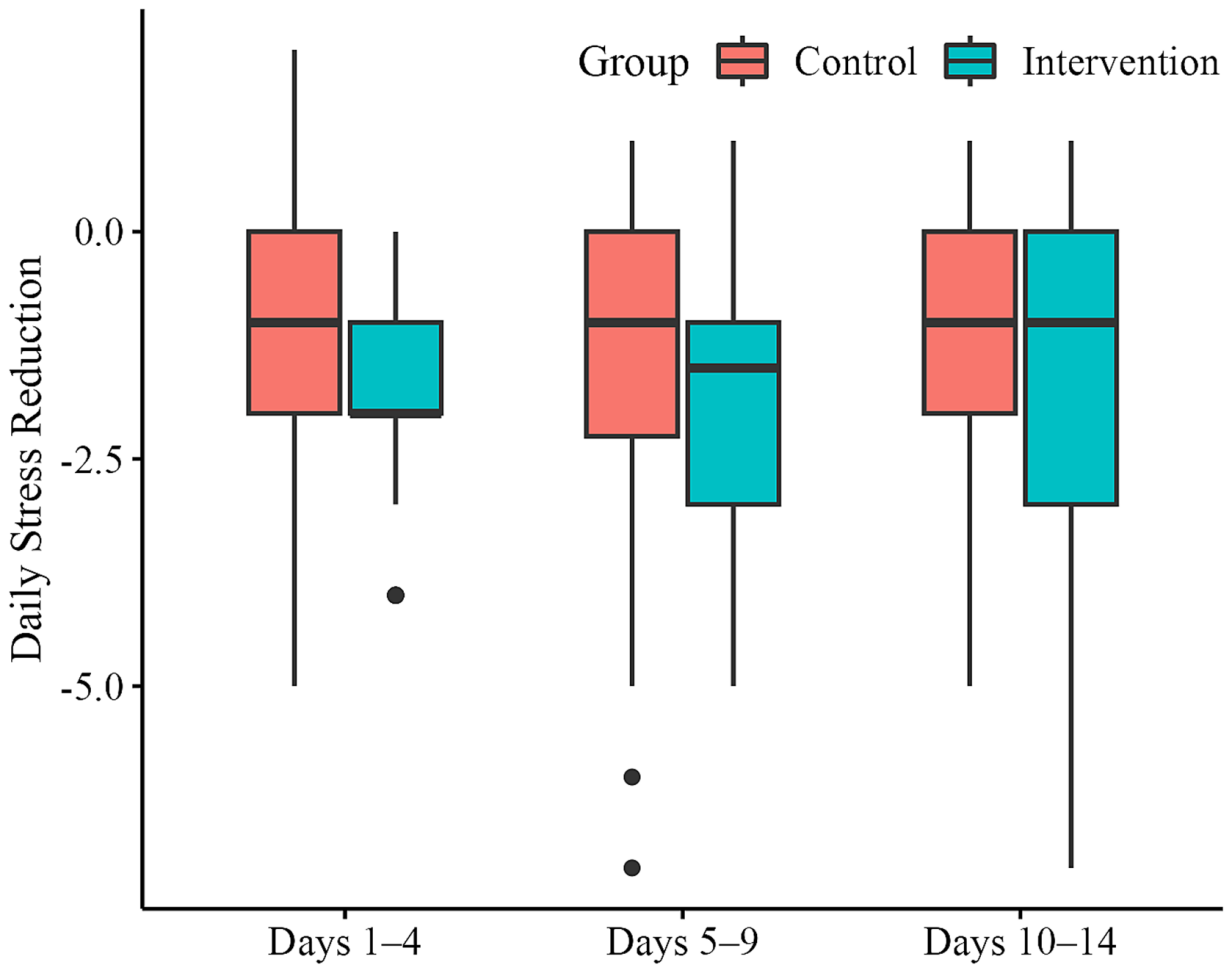
Mean daily stress reduction across the 14-day intervention period by group. Boxplots display within-session stress reduction (post-practice minus pre-practice stress ratings on a 1–10 scale) across three phases of the intervention: Days 1–4, Days 5–9, and Days 10–14. Negative values indicate stress reduction. The intervention group consistently showed larger daily stress reduction than the control group across the study period, especially during Days 1–9. Boxes represent interquartile range (IQR), horizontal lines indicate medians, whiskers extend to 1.5 × IQR, and circles represent outliers.

#### 3.5.2 Practice minutes

Practice logs were available for 32 of 35 participants (control *n* = 17, *M* = 425.8 min, *SD* = 282.8; intervention *n* = 15, *M* = 223.9 min, *SD* = 104.9), and total practice volume differed significantly between conditions (Wilcoxon rank sum test *U* = 199.5, *p* = 0.007). For each outcome we regressed the post-intervention score on its baseline value, condition, grand-mean-centered total practice minutes, and their interaction. For perceived stress, the Group × Minutes interaction was significant, *b* = −2.88, 95% CI [−6.67, −1.14], *p* = 0.014. Each additional 100 min of practice was associated with a 2.52-point lower post-intervention PSS-10 score in the intervention group, 95% CI [−4.57, −0.47], *p* = 0.018, whereas no association was observed in the control group, *b* = 0.37, 95% CI [−0.38, 1.12], *p* = 0.325. This interaction was robust to log and rank transformation of practice minutes, to restriction to the region of common support (≤ 500 min), to adjustment for covariates (Supplementary Tables S3, S4). Parallel analyses for mental well-being showed the same directional pattern but with an interval spanning zero, *b* = 2.14, 95% CI [−0.66, 4.94], *p* = 0.128. The slope was positive in the intervention group (2.40 points per 100 min, 95% CI [−0.21, 5.01], *p* = 0.070) and near zero in the control group (0.25, 95% CI [−0.68, 1.19], *p* = 0.582). No interaction emerged for nature connectedness, *b* = 0.87, 95% CI [−1.60, 3.34], *p* = 0.476. Because practice volume was self-selected rather than randomized and overlapped only partially between groups, these dose–response associations are correlational and should be treated as hypothesis-generating.

## 4 Discussion

This randomized controlled pilot study examined the effects of a brief, structured nature-based mindfulness program on mental well-being, perceived stress, and nature connectedness relative to an unstructured outdoor walking control. Three primary findings emerged: point estimates numerically favored the mindfulness intervention over walking alone for both mental well-being and perceived stress, but neither group difference reached statistical significance; both groups increased in nature connectedness without a significant between-group difference. Mediation analyses did not support nature connectedness as the mechanism driving these benefits.

These non-significant Time × Group interactions (mental well-being, *p* = 0.126; perceived stress, *p* = 0.147) should be interpreted alongside this pilot’s statistical power. Both outcomes showed high intraclass correlations (adjusted ICC = 0.72–0.74), reflecting substantial between-person stability that limits the sensitivity of a repeated-measures design this size: detecting effects of the observed magnitude with 80% power would require roughly 126–139 participants, three to four times our sample. Sensitivity analyses adjusting for baseline covariates yielded consistent point estimates (Supplementary Table S1), supporting the stability of the observed direction of effect. Consistent with this pilot’s aim, these estimates are best treated as preliminary input for powering a future trial.

Exploratory analyses indicated that longer individual sessions predicted greater momentary, self-reported stress relief regardless of group. Since the walking control group reported significantly more session minutes than the intervention group, this within-session benefit may reflect time spent outdoors generally rather than anything specific to the intervention. In contrast, only the intervention group showed a relationship between total accumulated session minutes and durable reduction in perceived stress over the two-week trial period. This may indicate that simply walking longer is unlikely to reproduce a lasting stress-reduction benefit, and that engagement with the intervention’s specific content may be the more plausible driver. As this dose–response pattern derives from self-selected, non-randomized practice time, it should be treated as hypothesis-generating and tested directly in a future study that experimentally manipulates practice dosage.

### 4.1 Mental well-being

This numerically favorable but non-significant pattern is consistent with prior work suggesting that the quality of engagement with nature, not merely time spent in it, predicts psychological benefit (Passmore and Holder, 2017). As noted previously, however, activity type and duration were not independently manipulated in this design; unequal recorded practice time may partly reflect the self-paced, unmonitored nature of the remote protocol rather than a clean quality-only comparison. We therefore treat this as a plausible, hypothesis-generating interpretation rather than a directly tested conclusion. Baseline well-being moderated the intervention’s effect on well-being: participants who began the study with lower well-being showed larger gains from the structured practice than those who began higher; among the latter the advantage over unstructured walking diminished and eventually reversed. This pattern is consistent with a ceiling on the scale and with the broader observation that psychological interventions tend to yield most where there is most room to move, but it also carries a substantive implication for this literature. However, this interpretation should be treated with caution as the interaction was not preregistered, and interaction effects estimated in samples of this size are known to be unstable and inflated in magnitude when first detected. The region of the baseline range in which the intervention advantage is clearly distinguishable from zero is also the region where the samples are limited. We regard the moderation as a hypothesis worth carrying into an adequately powered trial, ideally one that recruits deliberately across the well-being range rather than relying on whoever volunteers.

### 4.2 Stress reduction

Perceived stress as measured with the PSS-10 decreased in both conditions, with a larger raw decline in the intervention group (−4.13 points) than in the control group (−2.11 points). Although the Time × Group interaction was not statistically significant, the estimated between-group difference in change favored the intervention, *b* = −2.02, a pattern consistent with a larger effect size in the intervention group (*g* = −0.83 vs. −0.55 in control). A larger sample size is needed to confirm these findings. Consistent with the exploratory dose–response analysis (Section 3.5.2), total practice time was associated with a larger reduction in perceived stress only in the intervention group. As noted, this pattern is correlational rather than experimentally manipulated.

Daily practice logs, which capture session-level stress before and after each practice, showed the same pattern of no significant group difference (Section 3.5.1). Descriptively, the mean within-session reduction was similar in the early (1.56 points) and middle (1.56 points) portions of the trial and slightly smaller in the late portion (1.39 points); because the three phases were defined *post hoc*, were not stratified by condition, and were not formally compared, any suggestion of habituation or novelty effects should be treated as a hypothesis for future work.

This overall pattern is broadly consistent with SRT’s account of acute affective recovery through nature contact (Ulrich, 1983). However, our design cannot isolate SRT’s specific mechanisms or separate them from non-specific effects of study participation and repeated self-monitoring.

### 4.3 Nature connectedness

Both groups increased in nature connectedness over 2 weeks, but the between-group difference in magnitude of change was not statistically significant, and H3a was not supported. Exploratory mediation analyses further found no evidence that post-intervention nature connectedness mediated the intervention’s effects on stress or well-being. These null findings should not be interpreted as evidence against nature connectedness as a mechanism for nature-based interventions more broadly. Rather, they may reflect the brief two-week intervention window, the modest sample size, and the possibility that the CNS does not capture the specific form of engagement mindfulness practice cultivates—a possibility we consider further below.

The unexpected pattern may reflect differences in the type of engagement each condition cultivated. Existing connectedness measures such as the CNS (Mayer and Frantz, 2004) emphasize identity-based affiliation and emotional bond with nature as a whole, rather than the attentional intimacy or present-moment sensory contact cultivated through mindfulness. If mindful nature engagement fosters a qualitatively distinct mode of relating to the natural world, standard connectedness instruments may be insufficiently sensitive to detect it.

Future research should examine whether mindfulness-specific nature engagement indices, for example, scales assessing sensory awareness, present-moment attunement, or felt presence during nature contact, better capture the relational qualities that structured nature-based practices are designed to cultivate. It also remains possible that the null mediation result reflects the brevity of the present intervention rather than a genuine absence of a connectedness pathway: nature connectedness, as an identity-based construct, may require more sustained engagement than a two-week protocol allows to produce detectable change. Longer interventions, or those specifically designed to deepen felt identification with the natural world, may therefore yield a different pattern of results.

### 4.4 Practical implications

Although the present effects were modest and preliminary, the observed patterns are broadly consistent with prior work on brief mindfulness and nature-based interventions (Burdick and Camhi, 2024; Ibes and Forestell, 2022). The intervention’s relative simplicity represents a practical strength regardless of effect size: participants required only access to any outdoor space with natural elements, audio or video-guided instructions, and approximately 15 min per day. This low-barrier design is feasible across residential neighborhoods, campus green spaces, and urban parks, and lends itself to digital delivery at scale.

### 4.5 Limitations and future directions

As a pilot trial, our modest sample size (*N* = 35; *n* = 16 and *n* = 19 per group) limits the precision of our estimates, reflected in the wide confidence intervals around several key findings. Because these estimates were not statistically significant, they are not confirmatory and should be treated as hypothesis-generating for a larger, adequately powered trial.

Attrition was substantial: 66% of on boarded participants did not complete the trial. Completers and non-completers did not differ on baseline measures assessed. Intent-to-treat analysis suggests that the direction of both estimates was preserved across analyses, favoring the intervention condition on each outcome. However, confidence intervals included zero, and the attenuation under intent-to-treat analysis was of a similar relative magnitude for the two outcomes. We therefore report both as preliminary estimates, insufficient to establish an effect; this reporting is consistent with a pilot-trial framing. For planning a future confirmatory trial, the width of the intervals constitutes the most useful output. With a high attrition rate, both the maximum-likelihood and multiple-imputation estimates rest on a missing-at-random assumption that cannot be verified from the observed data, and at this level of missingness the imputed values are determined largely by the imputation model rather than by the participants themselves.

Although completers and non-completers did not differ on any measured baseline characteristics, unmeasured differences cannot be excluded, and the attenuation of the estimates when the full randomized baseline distribution is incorporated suggests that the observed-data analyses may modestly overstate the effect. Future trials should pursue more active retention strategies, including structured check-ins during the trial or app-based delivery that monitors engagement daily, along with pre-registered attrition protocols.

The sample was predominantly female (74.3%), White, and geographically concentrated in the Pacific Northwest, limiting generalizability to populations with different cultural relationships to nature or different access to green space; the region’s relatively high baseline nature access may itself have shaped intervention effects. The predominantly female composition of our sample (74.3%) may also limit generalizability to men: population-representative research finds men visit natural environments more frequently than women (Boyd et al., 2018). Evidence on nature connectedness itself is less consistent, with one cross-national study finding that women reported higher connection to nature despite lower rates of nature-based recreation participation (Rosa et al., 2020). We could not locate robust evidence on gender differences in mindfulness practices and note this as a gap in the existing literature. The age range of our sample (18–72, *M* = 46.32) skews middle-aged and may not generalize to younger adults. Participants were also self-selected volunteers, likely with some pre-existing interest in mindfulness or nature. Future studies should recruit more diverse and gender-balanced samples across race, age, and geography, and examine whether gender, prior mindfulness experience, urban versus rural setting, and baseline nature access moderate intervention effects.

The two-week intervention period also precluded examination of long-term maintenance, and the study relied exclusively on self-report; future work would benefit from objective measures such as cortisol or heart rate variability. Another limitation concerns our specific choice of nature connectedness measure. The CNS is designed as a trait measure, capturing a stable individual disposition rather than a state that is expected to shift over short periods. It remains possible that our null mediation findings partly reflect a mismatch between a trait-oriented instrument and a two-week intervention window, rather than a true absence of change in participants’ relationship to nature. A state-sensitive measure such as the Inclusion of Nature in Self scale (INS; Schultz, 2002), a single-item graphical measure that has been used specifically to detect momentary shifts in connectedness following brief nature-based interventions, may have been better suited to capture change across our short intervention period. Future studies using brief, repeated-measures nature-based designs should consider pairing a trait measure with a state-sensitive one to disentangle these possibilities. In addition, the use of another trait-scale, like the 6-item Nature Relatedness Scale (Nisbet et al., 2009) could provide new insights into the role of nature connectedness as a mediator for well-being effects in nature-based interventions.

A related design limitation concerns nature exposure duration. The intervention group reported substantially less total time in nature than controls. Activity type and duration were not independently manipulated, and unequal exposure time may partly reflect the self-paced, unmonitored nature of the remote protocol rather than a controlled comparison. Practice-minute data also reflect only self-reported time spent on the assigned activity itself, not participants’ total time outdoors that day; intervention participants may have taken additional unguided walks before or after their structured practice, and we did not monitor participants’ activity outside the study protocol. We caution against over-interpreting the duration comparison as a precise or complete accounting of relative nature exposure between groups. Future studies could address this directly by designing duration-matched conditions (e.g., equal-length structured mindfulness versus unstructured walking), and by adding an indoor mindfulness arm, which would allow a full factorial separation of the effects of setting (indoor versus outdoor) from the effects of structured mindfulness practice itself, an approach modeled by a recent pilot comparing indoor and outdoor mindfulness retreat settings (Djernis et al., 2021).

A further limitation concerns mechanism. This study was not designed to isolate what specifically drove the intervention’s effects. Improvements in the mindfulness group could reflect the mechanisms proposed by SRT or ART, specific mindfulness processes such as decentering, acceptance, or non-judgment, or more general factors such as relaxation, placebo effects, positive expectancy, increased self-monitoring through daily practice logging, or socially desirable responding. Our design cannot distinguish structured mindful engagement as a mindfulness-specific mechanism from the more general possibility that any structured, attention-directing activity, regardless of its specific mindfulness content, could produce similar benefits. We did not include measures of emotion regulation or decentering that might have helped distinguish among these possibilities. Future studies could incorporate such measures, include an active control condition matched on structure and self-monitoring but without mindfulness-specific content, or examine specific candidate mechanisms such as attentional broadening, reduced rumination, or increased sensory awareness.

### 4.6 Conclusion

This randomized controlled pilot study offers preliminary, non-confirmatory evidence that brief, structured mindfulness practices in natural settings may enhance mental well-being and produce a modest stress-reduction advantage beyond what is achieved through unstructured outdoor walking. Nature connectedness increased in both groups but did not serve as the mechanism through which the intervention produced its benefits, highlighting the need for more nuanced theoretical models of nature-mindfulness integration and more sensitive measures of human-nature relationships. Limitations mean that these findings should be treated as preliminary signals. Adequately powered trials with diverse samples, objective outcome measures, and experimentally controlled nature exposure doses are needed to test the robustness of these effects and clarify the mechanisms through which structured mindful engagement with nature supports psychological health.

## Data Availability

The raw data supporting the conclusions of this article will be made available by the authors, without undue reservation.
