# Supplementary material for: Does attention matter more than exposure? A pilot RCT of nature-based mindfulness for mental well-being and stress
Source: Front Psychol. 2026 Sep 24;17:1905841. doi: 10.3389/fpsyg.2026.1905841 (PMC13645504; doi:10.3389/fpsyg.2026.1905841)
Supplement: Supplementary file 1 [file Data_Sheet_1.PDF]

Supplementary Materials: Sensitivity Analyses

S.1 Covariate-Adjusted Models

Each primary model was refitted with age, prior nature exposure, and the baseline scores of the three scales other than the outcome added as fixed effects. For mental well-being the added baseline covariates were perceived stress, mindfulness and nature connectedness; for perceived stress they were mental well-being, mindfulness and nature connectedness; and for nature connectedness they were mental well-being, mindfulness and perceived stress. One participant did not report an age, so the adjusted models are fitted on 34 participants (68 observations) rather than 35 (70 observations).

For mental well-being and perceived stress the parameter of interest is the time × group interaction, which is the difference between arms in pre-to-post change. For nature connectedness the interaction was not significant ($\chi^2(1) = 0.44$, $p = .508$), so the parameter of interest there is the main effect of time in the additive model, that is the common pre-to-post change across both arms. Each parameter was tested by a likelihood-ratio test against the model omitting it, with both models fitted by maximum likelihood.

Table S1. Primary and covariate-adjusted estimates for the three outcomes

**Outcome**	**Parameter**	**Estimate**	**95% CI**	$\chi^2(1)$	***p***
Mental well-being	Time × Group				
Primary		2.63	[−0.94, 6.10]	2.34	.126
+ baseline covariates		3.12	[−0.29, 6.52]	3.25	.071
Perceived stress	Time × Group				
Primary		−2.02	[−4.84, 0.88]	2.11	.147
+ baseline covariates		−2.49	[−5.22, 0.23]	3.27	.071
Nature connectedness	Time				
Primary (additive)		4.14	[2.53, 5.79]	19.64	< .001
+ baseline covariates		4.29	[2.67, 5.92]	20.23	< .001

Note. Primary models $n = 35$ (70 observations); covariate-adjusted models $n = 34$ (68 observations), one participant missing age. Confidence intervals for the primary models are percentile bootstrap intervals (5,000 resamples); those for the covariate-adjusted models are Wald intervals computed as the estimate ± 1.96 *SE*. *p*-values are from likelihood-ratio tests of the stated parameter, with both models fitted by maximum likelihood. Mental well-being is measured by the WEMWBS, perceived stress by the PSS-10 on the 0–40 metric, and nature connectedness by the CNS. A negative interaction for perceived stress indicates a larger reduction in the intervention arm.

S.1.1 Mental well-being

Adjustment moved the interaction from 2.63 to 3.12 points, and the likelihood-ratio *p*-value from .126 to .071. The direction and approximate magnitude of the estimate are therefore unchanged, and adjustment does not attenuate it. The estimate does not reach conventional significance in either specification, so what the analysis supports is that the primary estimate is not an artefact of baseline imbalance, not that a significant effect has been confirmed. Of the covariates, baseline perceived stress ($b = -0.53$, $SE = 0.19$) and prior nature exposure ($b = -1.56$, $SE = 0.65$) were the strongest predictors of well-being.

S.1.2 Perceived stress

Adjustment moved the interaction from −2.02 to −2.49 points, with the likelihood-ratio *p*-value falling from .147 to .071. As with well-being, the estimate is stable in sign and grows slightly in magnitude under adjustment, and remains non-significant in both specifications. Baseline mental well-being was a strong negative predictor of stress ($b = -0.43$, $SE = 0.12$), consistent with the −.59 baseline correlation between the two scales.

S.1.3 Nature connectedness

The pre-to-post increase in nature connectedness was 4.14 points in the primary additive model and 4.29 points with covariates added, and was significant in both ($p < .001$). This is the one outcome for which a significant effect is present, and it is unaffected by adjustment. It should be read as a change over time common to both arms rather than an effect of the intervention, since the time × group interaction was not significant.

S.1.4 Summary

Across all three outcomes, adding baseline covariates left the parameter of interest unchanged in sign and similar in magnitude, and in no case reversed a conclusion. For mental well-being and perceived stress the adjusted estimates are slightly larger than the unadjusted ones, so the primary analysis is if anything the more conservative of the two.

S.2 Mediation Sensitivity Analysis

The exploratory mediation analyses ask whether the effect of allocation on perceived stress, and on mental well-being, operates through post-trial nature connectedness. Both were estimated with nonparametric bootstrap percentile intervals (5,000 resamples), adjusting for the baseline value of the mediator, the baseline value of the outcome, age and prior nature exposure, on the 34 participants with complete covariate data.

Table S2. Causal mediation estimates with nature connectedness as mediator

Outcome	Effect	Estimate	95% CI	*p*
Perceived stress	Total effect (c)	−2.11	[−4.33, 0.65]	.136
	Direct effect (c′)	−2.20	[−4.77, 0.61]	.124
	Indirect effect (ACME)	0.10	[−0.97, 1.81]	.779
	Proportion mediated	−0.05	[−2.15, 1.41]	.834
Mental well-being	Total effect (c)	2.46	[−1.65, 6.55]	.282
	Direct effect (c′)	2.66	[−1.03, 6.83]	.188
	Indirect effect (ACME)	−0.20	[−2.41, 1.41]	.724
	Proportion mediated	−0.08	[−4.63, 4.18]	.934

Note. $n = 34$, 5,000 bootstrap resamples. ACME = average causal mediation effect. The mediator is post-trial nature connectedness. Models adjust for baseline nature connectedness, the baseline value of the outcome, age and prior nature exposure. No effect in this table is statistically significant.

No indirect effect was detected for either outcome, and the proportion mediated is estimated so imprecisely that its interval spans implausible values in both directions. This alone does not establish the absence of mediation, since the mediator–outcome relationship is not randomized and could be confounded. We therefore applied the sensitivity analysis of Imai, Keele and Yamamoto (2010), which asks how strongly the residuals of the mediator and outcome models would have to be correlated (ρ) for the estimated indirect effect to equal zero.

The indirect effects are fragile: a mediator–outcome residual correlation of only $\rho = \pm 0.30$ is enough to move the estimated indirect effect to zero. Because the estimated indirect effects are already indistinguishable from zero, the result shows that the mediation estimate carries almost no information at this sample size. A confounder within the range of plausibility would move it in either direction.

The direct effects are considerably more robust. Across the whole range searched, $|\rho| \leq 0.90$, the estimated direct effect never crosses zero for either outcome: it stays between −1.60 and −2.80 for perceived stress, and between 1.46 and 3.87 for mental well-being. The conclusion supported by these analyses is therefore that whatever difference exists between groups is not detectably transmitted through nature connectedness, and that a larger sample would be needed to estimate any indirect effect with useful precision.

S.3 Practice Volume and Outcomes

Total practice minutes were available for 32 of the 35 participants; three did not return a practice log. Practice volume differed substantially between arms (control $M = 425.8$, $SD = 282.8$; intervention $M = 223.9$, $SD = 104.9$; Mann–Whitney $U = 199.5$, $p = .007$), and the two distributions overlap only between 180 and 500 minutes, a range containing 22 of the 32 participants. Every participant above 500 total minutes is in the control group. Any group × minutes interaction is therefore identified principally on the overlapping range, and Section B.3.2 refits the model restricted to it.

For each outcome the post-intervention score was regressed on its baseline value, group, total practice minutes in 100-minute units centred at the grand mean, and the group × minutes interaction. Control is the reference level, so the minutes coefficient is the slope within the control group, and the interaction is the difference between groups in that slope. The intervention slope was obtained by refitting with intervention as the reference.

Table S3. Practice volume as a predictor of post-intervention outcomes, by group

Outcome	Parameter	Estimate	95% CI	p
Perceived stress	Minutes slope, control	0.37	[−0.38, 1.12]	.325
	Minutes slope, intervention	−2.52	[−4.57, −0.47]	.018
	Group × Minutes	−2.88	[−5.13, −0.64]	.014
Mental well-being	Minutes slope, control	0.25	[−0.68, 1.19]	.582
	Minutes slope, intervention	2.40	[−0.21, 5.01]	.070
	Group × Minutes	2.14	[−0.66, 4.94]	.128
Nature connectedness	Minutes slope, control	0.21	[−0.58, 0.99]	.592

Outcome	Parameter	Estimate	95% CI	p
	Minutes slope, intervention	1.08	[−1.20, 3.36]	.342
	Group × Minutes	0.87	[−1.60, 3.34]	.476

Note. $n = 32$. Estimates are outcome points per additional 100 minutes of total practice. Confidence intervals and *p*-values are from Wald test with the baseline value of the outcome as a covariate. Confidence intervals can be slightly different than the main text, which is from bootstrapping. The Group × Minutes row is the difference between the two slopes above it. Perceived stress is on the PSS-10 0–40 metric, so a negative slope indicates lower stress at higher practice volume; for mental well-being and nature connectedness a positive slope is the favourable direction.

S.3.1 Robustness of the perceived-stress interaction

Table S4. Group × Minutes interaction for perceived stress across specifications

Specification	n	Estimate	95% CI	p
Primary (per 100 minutes)	32	−2.88	[−5.13, −0.64]	.014
Log total minutes	32	−8.14	[−14.44, −1.85]	.013
Rank-transformed minutes	32	−0.47	[−0.82, −0.11]	.012
Average minutes per day	32	−0.34	[−0.66, −0.03]	.036
Excluding highest-leverage case	31	−4.42	[−7.61, −1.23]	.009
Excluding largest practice total	31	−3.01	[−5.40, −0.62]	.016
Restricted to common support (≤ 500 min)	27	−3.91	[−7.37, −0.46]	.028
Common support, log minutes	27	−10.08	[−19.58, −0.58]	.039
Group-specific baseline slopes	32	−2.75	[−5.05, −0.45]	.021
Adding age, nature exposure, solitary practice	31	−2.34	[−4.66, −0.02]	.048

Note. Estimates for the log and rank specifications are on those transformed scales and are not comparable in magnitude with the per-100-minute rows. The two excluded cases are the participant with the highest leverage (500 minutes, intervention arm) and the participant with the largest practice total (1,260 minutes, control arm); each was removed separately, not together. The final row adds age, prior nature exposure and proportion of sessions practised alone, and loses one participant who did not report an age.

The interaction is stable in direction and significant level across all ten specifications, and dropping either of the two most influential participants increases rather than reduces its magnitude. A leave-one-out refit across all 32 participants gave interaction *p*-values between .004 and .055, retaining significance at the .05 level in 31 of the 32 fits. Restricting the sample to the region of common support, which discards the five control participants above 500 minutes, likewise strengthens the estimate.

These two null results carry little information. With 32 participants and five model parameters, the smallest interaction detectable at 80% power is $f^2 = 0.29$, corresponding to $\Delta R^2 \approx .23$, which is a large effect on Cohen's benchmarks. Achieved power was .75 for the perceived-stress interaction, .35 for mental well-being and .11 for nature connectedness. The mental well-being and nature connectedness results should therefore be interpreted as inconclusive rather than as evidence that practice volume is unrelated to those outcomes.

No correction for multiple comparisons was applied, consistent with the exploratory framing. For reference, applying a Benjamini–Hochberg correction across the three interaction tests leaves the

perceived-stress result below the conventional threshold (adjusted $p = .041$); the other two are unaffected in substance.

Reference: Imai, K., Keele, L., & Yamamoto, T. (2010). Identification, inference and sensitivity analysis for causal mediation effects. *Statistical Science, 25*(1), 51–71.
