# Supplementary material for: Does attention matter more than exposure? A pilot RCT of nature-based mindfulness for mental well-being and stress
Source: Front Psychol. 2026 Sep 24;17:1905841. doi: 10.3389/fpsyg.2026.1905841 (PMC13645504; doi:10.3389/fpsyg.2026.1905841)
Supplement: Supplementary file 2 [file Supplementary_file_1.pdf]

Supplementary Material.

Guided nature-based mindfulness practice session script excerpts.

Nature-Based Mindfulness Program: Script Excerpts (Research Version)

The following excerpts are drawn from the two guided practice sequences described in section 2.2.1 and are provided to support methodological transparency and replication. Across both sequences, the practices integrated recurring elements of bodily slowing and grounding, multisensory engagement with natural elements, embodied breathing and imagery emphasizing human–nature reciprocity, and brief reflective exercises fostering relaxation, awe, and nature connectedness.

Excerpt 1: Bodily Slowing and Grounding

> "As you walk to your practice location, deliberately slow your pace to about half the speed you usually walk. Allow your body to soften as you move. Breathe in through your nose, filling your lungs and belly, and exhale fully through your mouth. Notice how slowing your body begins to quiet your mind."

Excerpt 2: Multisensory Awareness

> "Bring your attention to the sounds around you. Listen without labeling, simply notice near and distant sounds. Then shift your awareness to your sense of smell. What does the air carry in this place? Finally, reach out and touch a natural object nearby. Notice its texture, temperature, and shape."

Excerpt 3: Embodied Imagery and Human–Nature Reciprocity

“Place your hands on the trunk of a tree and close your eyes. Imagine how the tree receives your exhale and returns it as oxygen for your next inhale. With each breath, sense this quiet exchange between your body and the living world around you.”

Excerpt 4: Reflective Engagement with Nature

“Imagine you are walking through an art gallery created by nature. Pause in front of one ‘work of art’, a patch of sunlight, a leaf, a pattern in the bark. Take a moment to appreciate its beauty, knowing it will never appear in exactly this way again.”
