# Supplementary material for: Does attention matter more than exposure? A pilot RCT of nature-based mindfulness for mental well-being and stress
Source: Front Psychol. 2026 Sep 24;17:1905841. doi: 10.3389/fpsyg.2026.1905841 (PMC13645504; doi:10.3389/fpsyg.2026.1905841)
Supplement: Supplementary file 3 [file Table_1.pdf]

Supplementary Table Appendix.

Participant characteristics and model estimates (Tables A1–A5).

Table A1

Participant Demographic Characteristics by Group

Characteristic	Intervention (n = 16)	Control (n = 19)
Age (years)		
M (SD)	41.73 (15.71)	49.95 (18.50)
Range	19–62	18–72
Gender		
Female	11 (68.8%)	15 (78.9%)
Male	2 (12.5%)	3 (15.8%)
Transgender	2 (12.5%)	0 (0.0%)
Non-binary	1 (6.3%)	1 (5.3%)
Ethnicity		
Asian	0 (0.0%)	3 (15.8%)
Hispanic	1 (6.3%)	0 (0.0%)
Caucasian	14 (87.5%)	14 (73.7%)
Not reported	1 (6.3%)	2 (10.5%)

Note. N = 35. Values represent frequency counts with percentages in parentheses. One participant in the intervention group was missing age data.

Table A2

Baseline comparison of completers (n = 35) and non-completers (n = 68) among randomized participants with intake data (N = 103).

Baseline measure	Completers	Non-completers	Test Statistics	df	p	p (Holm)	Hedges g	n used
Mental well-being (WEMWBS)	49.29	48.38	t = 0.59	101	.554	1.000	0.12	35 / 68
Perceived stress (PSS-10)	16.71	18.43	t = −1.47	101	.145	1.000	−0.30	35 / 68
Nature connectedness (CNS)	55.49	57.00	t = −0.96	101	.341	1.000	−0.20	35 / 68
Trait mindfulness (FFMQ-SF)	50.69	52.16	t = −0.89	101	.374	1.000	−0.18	35 / 68
Age (years)	46.32	44.24	t = 0.58	100	.563	1.000	0.12	34 / 68
Typical nature exposure	4.63	4.68	t = −0.12	101	.906	1.000	−0.02	35 / 68
Mindfulness practice frequency	2.77	3.06	t = −0.91	77	.365	1.000	−0.22	26 / 53
Any prior mindfulness practice	—	—	χ^2 = 0.03	1	.865	1.000	—	35 / 68
Gender	—	—	χ^2 = 4.46	4	.347	1.000	—	35 / 68
Ethnicity	—	—	χ^2 = 4.50	6	.610	1.000	—	35 / 68

Note. Tests as in Section 2.4; p (Holm) is Holm–Bonferroni-adjusted across the ten comparisons. n used gives completers/non-completers per comparison: age was missing for one completer, and mindfulness practice frequency was asked only of participants reporting a regular practice. PSS-10, CNS, and FFMQ-SF are reverse-scored where applicable; self-rated green space is omitted (undefined for non-practicers). No comparison was significant before or after adjustment (largest |g| = 0.30).

Table A3

Descriptive Statistics for All Outcomes by Group and Time Point

Outcome / Group	*n*	Pre-Test *M* (*SD*)	Post-Test *M* (*SD*)	Change *M*
Mental Well-Being (WEMWBS)				
Intervention	16	47.00 (6.43)	51.31 (5.49)	+4.31
Control	19	51.21 (7.33)	52.89 (8.54)	+1.68
Perceived Stress (PSS-10)				
Intervention	16	17.69 (5.13)	13.56 (4.99)	−4.13
Control	19	15.89 (5.90)	13.79 (6.00)	−2.11
Nature Connectedness (CNS)				
Intervention	16	56.25 (6.55)	59.81 (6.12)	+3.56
Control	19	54.84 (5.71)	59.47 (5.52)	+4.63

Note. $N = 35$. WEMWBS = Warwick–Edinburgh Mental Well-Being Scale (possible range 14–70); PSS-10 = Perceived Stress Scale (possible range 0–40); CNS = Connectedness to Nature Scale (possible range 14–70). Higher WEMWBS and CNS scores indicate greater well-being and nature connectedness respectively; higher PSS-10 scores indicate greater perceived stress. Change = post-test minus pre-test. No outcome data were missing, so *n* is the same at both time points. All scales are scored with the negatively worded items reversed in accordance with their published scoring manuals (PSS-10 items 4, 5, 7, and 8; CNS items 4, 12, and 14; WEMWBS has no reverse-scored items), and PSS-10 totals are reported on the standard 0–4 item metric.

Table A4

Linear Mixed-Effects Results and Follow-Up Effect Sizes for All Outcomes

Outcome	Estimate / Hedges' *g*	Std. Error	*t*	*p*	95% CI
Mental Well-Being (WEMWBS)					
Time (control group)	1.684	1.176	1.432	—	[−0.59, 4.09]
Time × Group	2.628	1.740	1.511	.126	[−0.94, 6.10]
Group	−4.211	2.418	−1.741	—	[−8.87, 0.60]
Time (both groups)	2.886	0.883	3.269	.002	[1.15, 4.59]
Intervention (pre–post)	0.67			—	[0.14, 1.19]
Control (pre–post)	0.38			—	[−0.07, 0.83]
Perceived Stress (PSS-10)					
Time (control group)	−2.105	0.955	−2.205	—	[−4.06, −0.26]
Time × Group	−2.020	1.412	−1.430	.147	[−4.84, 0.88]
Group	1.793	1.887	0.950	—	[−2.00, 5.59]
Time (both groups)	−3.029	0.714	−4.241	< .001	[−4.41, −1.62]
Intervention (pre–post)	−0.83			—	[−1.38, −0.27]
Control (pre–post)	−0.55			—	[−1.01, −0.08]
Nature Connectedness (CNS)					
Time (control group)	4.632	1.121	4.130	—	[2.34, 6.80]
Time × Group	−1.069	1.658	−0.645	.508	[−4.38, 2.33]
Group	1.408	2.021	0.697	—	[−2.65, 5.45]
Time (both groups)	4.143	0.819	5.058	< .001	[2.53, 5.79]

Outcome	Estimate / Hedges' *g*	Std. Error	*t*	*p*	95% CI
Intervention (pre–post)	0.58			—	[0.07, 1.09]
Control (pre–post)	1.11			—	[0.54, 1.66]

Note. N = 35 (16 intervention, 19 control), 70 observations. Time is coded 0 = pre-test, 1 = post-test; Control is the reference group. Time is the control group's pre–post change; Time (both groups) is the common pre–post change from the reduced additive model. P values are likelihood-ratio tests as described in Section 2.4; Time (control group) and Group were not tested (—). CIs use 5,000 bootstrap resamples; Hedges' g CIs are noncentral t.

Table A5

Estimated group difference in pre-to-post change (intervention minus control) under maximum-likelihood, and multiple-imputation analyses.

Outcome	Analysis	n	Estimate	SE	95% CI
Mental well-being					
	Maximum likelihood, all randomized	103	1.96	1.63	[−1.24, 5.16]
	Multiple imputation (m = 500)	103	1.50	1.86	[−2.16, 5.15]
Perceived stress					
	Maximum likelihood, all randomized	103	−1.63	1.32	[−4.21, 0.95]
	Multiple imputation (m = 500)	103	−1.35	1.46	[−4.22, 1.52]
Nature connectedness					
	Maximum likelihood, all randomized	103	−1.21	1.59	[−4.33, 1.92]
	Multiple imputation (m = 500)	103	−1.75	1.81	[−5.31, 1.80]

Note. Estimates are the Time × Group interaction from a linear mixed-effects model with a random intercept for participant, fitted by full-information maximum likelihood, and under multiple imputation (500 datasets, missing-at-random, Rubin's rules) as described in Section 2.4. WEMWBS and CNS are scored so that higher values indicate greater well-being and connectedness; PSS-10 is scored so that higher values indicate greater perceived stress.
